# The Presence of Immature GV− Stage Oocytes during IVF/ICSI Is a Marker of Poor Oocyte Quality: A Pilot Study

**DOI:** 10.3390/medsci8010004

**Published:** 2020-01-16

**Authors:** Pia Astbury, Goutham N. Subramanian, Jessica Greaney, Chris Roling, Jacqui Irving, Hayden A. Homer

**Affiliations:** 1 Christopher Chen Oocyte Biology Research Laboratory, UQ Centre for Clinical Research, The University of Queensland, Herston 4029, Queensland, Australia; 2 Queensland Fertility Group, Brisbane 4000, Queensland, Australia; 3 Reproductive Endocrinology & Infertility Clinic, Royal Brisbane & Women’s Hospital, Brisbane 4029, Queensland, Australia

**Keywords:** immature oocyte, oocyte, DNA damage, oocyte quality, IVF

## Abstract

Here we investigate whether the presence of germinal vesicle-stage oocytes (GV− oocytes) reflects poor oocyte developmental competence (or quality). This was a prospective, non-randomised, cohort pilot-study involving 60 patients undergoing in vitro fertilization/ intracytoplasmic sperm injection for whom complete pregnancy outcome data were available. Patients in whom GV− oocytes were retrieved (GV+) at transvaginal oocyte retrieval (TVOR) were compared with those from whom no GVs were retrieved (GV−). We found that GV+ (*n* = 29) and GV− (*n* = 31) patients were similarly aged (35.4 vs. 36.4 years; *p* = 0.446). GV+ patients had a mean of 2.41 ± 2.03 GVs and comparable yields of MII oocytes to GV− patients (11 ± 6.88 vs. 8.26 ± 4.84; *p* = 0.077). Compared with GV− patients, GV+ patients had markedly lower implantation rates (11.8% vs. 30.2%; *p* = 0.022) as well as oocyte utilisation rates for clinical pregnancy (2.3% vs. 6.8%; *p* = 0.018) and live-birth (1.9% vs. 5.7%; *p* = 0.029). DNA damage levels measured using γH2AX immunostaining were not different in oocytes from women <36 years versus those ≥36 years (*p* = 0.606). Thus, patients who have GV− stage oocytes at TVOR exhibit poor oocyte quality reflected in reduced per-oocyte pregnancy success rates and uniformly high levels of oocyte DNA damage.

## 1. Introduction

Oocytes ovulated by women in their late 40′s would have been arrested in primordial follicles for a staggering 4–5 decades. During this protracted arrest, the oocyte’s DNA is prone to damage from exogenous environmental toxins as well as endogenous reactive oxygen species (ROS), which accumulate with advancing age in parallel with declining mitochondrial function [1,2,3,4,5]. Vulnerability to damage is further exacerbated by a decline in DNA repair capacity as oocytes age [2,4]. Thus, DNA damage in oocytes is a molecular readout of quality or developmental competence, which is defined as the capacity to support meiotic maturation, fertilisation, embryonic development and successful pregnancy [6].

Following hCG trigger during stimulated IVF cycles, typically around 70–85% of oocytes obtained at transvaginal oocyte retrieval (TVOR) are mature (MII-arrested) eggs with the other 15–30% being at MI- and GV− stages [7,8,9,10]. It is not known why some oocytes remain GV− arrested despite being exposed to an hCG stimulus. One possibility is that these oocytes are derived from small follicles in which the LH-signalling mechanism has not fully developed. This is unlikely to fully explain their occurrence, however, since mature eggs can be retrieved from around 40% of follicles as small as 3–10 mm in diameter [11]. Ovarian hyper-response might also predispose to the occurrence of immature oocytes [12], albeit this has not been a consistent finding [13]. Another possibility is that GV− oocytes occur due to a more widespread defect that prevents only a fraction of oocytes from undergoing maturation; DNA damage in oocytes is one plausible mechanism by which this could occur [14]. If the latter model were true, an important question that then arises is, how might such a defect impact the MII-oocytes arising out of the same cohort?

It remains unresolved whether GV− oocytes are an independent occurrence unrelated to the remaining cohort of oocytes or it may represent a wider pathophysiological process of which GV− oocytes are a by-product. We reasoned that if the former were true, then in cycles with GVs, companion MII oocytes should produce similar outcomes to MII oocytes from cycles without GVs. Here, for the first time that we are aware of, we sought to test this by prospectively investigating how the presence of GV− oocytes impacts the per-oocyte and per-embryo performance of companion MII oocytes and quantifying DNA damage levels in GV− oocytes from these cycles. Our data based on clinical pregnancy outcomes and oocyte DNA damage levels suggest that the occurrence of GV− stage oocytes may be a surrogate marker for poor oocyte quality.

## 2. Materials and Methods

### 2.1. Subjects

All patients involved in the study provided written informed consent prior to starting ovarian stimulation. GV− oocytes were donated by consenting patients undergoing IVF/ICSI with the Queensland Fertility Group between February 2017 and July 2018. The study included only one round of ovarian stimulation per patient. Patients were excluded if they were undergoing fertility preservation or donating oocytes. Following consent to participate in the study, patients were followed up when oocytes were denuded of surrounding cumulus cells for determining maturation status (ICSI patients) or fertilisation (IVF patients).

The study was approved by the Queensland Fertility Group Human Research Ethics Committee (QFG14.16).

### 2.2. Ovarian Stimulation Protocol

Ovarian stimulation regimes were based on an antagonist protocol. Typically, either recombinant follitropin alfa (Gonal-F; Merck Serono Australia Pty Ltd., Sydney, Australia) or recombinant follitropin beta (Puregon; Merck Sharp & Dohme Australia Pty Limited, Sydney, Australia) was used for stimulating follicular development. GnRH antagonist, Ganirelix acetate (Orgalutran; Merck Sharp & Dohme, Sydney, Australia), was introduced on Day 5 of FSH to prevent a premature LH surge. Monitoring was undertaken using transvaginal scanning and serum measurements of oestradiol, progesterone and LH. When 2–3 follicles were present at 17–18 mm in diameter, oocyte maturation was triggered using recombinant human chorionic gonadotrophin (Ovidrel; Merck Serono Australia Pty Ltd., Sydney, Australia). TVOR was performed under general anaesthetic 36–37 h following the trigger. Only follicles >14 mm in diameter were aspirated using a 17G double lumen ovum aspiration needle (K-OPSD-1730-A-L; Cook Australia Pty Ltd., Brisbane, Australia) under transvaginal ultrasound guidance. 

Following retrieval and insemination, oocytes were cultured in G-IVF media (Vitrolife, Gothenburg, Sweden). Pronuclear stage embryos were then cultured in single-step G-TL media (Vitrolife). Embryo transfers were performed at either cleavage-stage (Day 2/3) or blastocyst-stage (Day 5/6) using a Guardia^TM^ Access soft catheter (K-JETS-7019; Cook Australia Pty Ltd., Brisbane, Australia). Luteal support was with vaginal progesterone (Crinone 8% Vaginal Gel; Merck Serono, Sydney, Australia) commencing on the day following TVOR.

Quantitative serum beta-hCG levels were performed 10–12 days following embryo transfer and if >10 IU/mL, repeated 1 week later. Patients with rising serum beta-hCG levels had a transvaginal pregnancy scan performed 2–3 weeks later. Surplus embryos of suitable grade were cryopreserved using a commercial vitrification kit (Rapid-i™ Kit; Vitrolife, Gothenburg, Sweden).

### 2.3. Human Oocytes

Human oocytes were denuded of surrounding cumulus cells by means of mechanical pipetting using a 140 µm Flexipet (Cook Australia Pty Ltd., Brisbane, Australia) in combination with pharmaceutical grade hyaluronidase (Hyalase^®^; Sanofi-Aventis Australia Pty Ltd., Brisbane, Australia) reconstituted in pH-stabilised G-MOPS media (Vitrolife, Gothenburg, Sweden). GV− oocytes were identified by the presence of a clearly defined GV containing the typical prominent nucleolus. MII-stage oocytes were identified by the presence of the first polar body, while oocytes were assigned as MI-stage if they lacked both a GV and a polar body. Following identification of GV− oocytes, embryologists informed investigators who then immediately transferred oocytes to the Oocyte Research Lab in G-MOPs media under mineral oil within a pre-warmed transport incubator (Thermo Cell Transporter 3018; Labotect, Rosdorf, Germany) for immediate fixation (see below). All embryologists were educated about the study beforehand and the need to inform investigators of the presence of GV− oocytes as soon as feasible following identification.

### 2.4. Clinical Outcomes

Total numbers of GV−, MI- and MII-stage oocytes were determined for each patient’s cycle. Fertilisation was calculated as the numbers of zygotes with two pronuclei per inseminated MII oocyte at fertilisation checked the day following TVOR.

Because we were principally interested in the quality of individual oocytes, we sought measures of per-oocyte performance. Hence, the principal outcomes analysed were the number of usable embryos (defined as before as embryos deemed suitable for either fresh transfer or for cryopreservation [15]), clinical pregnancy and live-birth rates per MII oocyte, this ratio being referred to as oocyte utilisation rates (in essence, oocyte utilisation rates provided a measure of the proportion of MIIs that produced either clinical pregnancies or live-births) [15,16]. Moreover, since embryo quality is predominantly a function of oocyte quality, per-embryo performance was assessed using implantation rates and live-birth rates per embryo transferred. Clinical pregnancy rates were based on numbers of intrauterine gestation sacs with a positive fetal heartbeat. Implantation rate was defined as the number of gestational sacs with a positive fetal heartbeat divided by the total number of embryos transferred. Because we wanted to evaluate the performance of utilised MII oocytes, but not all usable embryos were transferred, we derived an estimate of the number of oocytes that had been utilised per patient (Corrected MII oocytes) using the formula:

Corrected MII oocytes = (Total embryos used/Total usable embryos) × Total MII oocytes(1)

### 2.5. Immunostaining of GV− Oocytes and Quantification of DNA Damage

Human oocytes were fixed and immunostained using protocols as described in detail and validated extensively previously [17,18,19,20,21]. Briefly, oocytes were washed in PIPES, HEPES, EGTA and Magnesium Sulphate (PHEM) buffer (pH 7.0) and pre-permeabilised in 0.25% Triton-X in PHEM. Oocytes were then fixed in 3.7% paraformaldehyde solution in PHEM for 20 min. Oocytes were blocked overnight in 3% BSA in PBS containing 0.05% Tween-20 at 4 °C. Primary antibody incubation with anti-gamma H2A.X antibody (Abcam-ab11174; 1:200) was carried out for 1 h at 37 °C. Following three 5-min washes in phosphate-buffered saline (PBS) containing 0.5% BSA and 0.05% Tween-20, oocytes were incubated with the appropriate Alexa Fluor 488-conjugated secondary antibodies (1:200; Thermo Fisher, Melbourne, Australia) for 1 h at 37 °C. 

Imaging was performed using a Leica TCS SP8 confocal microscope (Leica Microsystems, Wetzlar, Germany) equipped with a 20×/0.75 NA Apochromat water-immersion objective; Super Z-Galvo stage for ultra-rapid movement in the z-plane with extreme precision; as well as 405 nm, 458 nm, 488 nm, 514 nm, 561 nm and 633 nm laser lines as described previously [22]. Oocytes were imaged in 1–2 μL micro-drops of PBS containing 0.5% BSA in glass-bottom dishes (35 × 10 mm dish, no. 0 coverslip; MatTek) under mineral oil. Automated image capture was driven by Leica LAS X software (Version: Lifescience 3.7.1, Leica Microsystems, Germany). For each oocyte, the positions of GV in the z-axis were located and a complete stack was then derived by setting the upper and lower limits to capture the entire GV. Z-stacks were acquired with step intervals of 1–2 μm at a speed of 600 Hz. Images were imported from LAS X and analysed using Image J software (Version: 1.51s, NIH, Maryland, USA). The region enclosing the GV was specified as ‘region of interest’ (ROI) and an automated ‘Object Counter 3D’ plugin was used to count the number and size of γH2AX foci across a defined confocal stack covering the entire GV region. γH2AX foci were considered as signals exceeding a 25-voxel threshold using the threshold feature on the Object Counter 3D plugin.

### 2.6. Statistical Analyses

GraphPad Prism (Version 8.0.1, GraphPad, San Jose, CA, USA) was used to calculate mean ± standard deviation (SD) and proportions with 95% confidence intervals (CI). Statistical comparisons were made using the two-tailed Student’s *t*-test for continuous data and the Chi-squared test for categorical data. Graphs were prepared in GraphPad. A *p* value < 0.05 was considered statistically significant.

## 3. Results

### 3.1. Overall Characteristics of Study Population and of Treatment Cycles

Sixty patients undergoing either in vitro fertilization (IVF; *n* = 14) or intracytoplasmic sperm injection (ICSI; *n* = 46) with a mean age of 36 ± 4.7 (range 24.7–45.8 years) took part in the study (Table 1). Both groups were broadly similar in terms of their infertility problems (Table 2). No study patient underwent ovarian stimulation without having at least one embryo transferred. Of these, 29 (48.3%) had GV− oocytes retrieved (GV+) and 31 (51.7%) lacked GV− oocytes (GV−). The proportions of patients having IVF and ICSI were similar in the two arms (*p* = 0.22) with the majority having ICSI in both groups (83% and 71%) (Table 1). The mean age of GV+ patients was not different from that of GV− patients (36.4 versus 35.4 years; *p* = 0.45). 

A mean of 1.11 ± 0.31 and 1.08 ± 0.27 embryos were transferred in the GV+ and GV− groups, respectively (*p* = 0.597) with a single embryo being transferred in the overwhelming majority of cycles (89.13% for GV+ and 92.5% for GV− patients; *p* = 0.435) (Table 1). There was no difference in embryonic stage at transfer; 78.4% and 83.7% blastocyst-stage transfers for GV+ and GV−, respectively (*p* = 0.441) (Table 1).

In the GV+ group, 23 of 29 patients (79.3%) utilised all the embryos produced from a single round of stimulation, either in a single fresh or in a fresh followed by subsequent thaw cycles, similar to the GV− group (23 of 31 patients; 74.2%; *p* = 0.435) (Table 1). In most cases, unutilised embryos occurred because patients had had a live birth before utilising all cryopreserved embryos (7 of 8 [87.5%] GV− patients and 4 of 6 [66.67%] GV+ patients). One GV+ patient did not utilise all embryos in the study cycle because she went on to have a live-birth in a subsequent stimulated cycle. Only one patient in each group who currently has unutilised frozen embryos has not had a live-birth. 

A total of 675 oocytes were retrieved from GV+ and GV− patients of which, 575 (85.2%) were MII-stage, 30 were MI-stage (4.4%) and 70 (10.4%) were GVs (Table 3). A total of 319 and 256 MII oocytes were collected in the GV+ group and GV− group, respectively, with comparable mean numbers for both groups (11.0 ± 6.88 vs. 8.26 ± 4.84; *p* = 0.078). 

### 3.2. Clinical Outcomes for GV+ and GV− Patients

To estimate oocyte quality, we sought measures of per-oocyte and per-embryo performance rather than per-cycle outcomes, since per-cycle results are also influenced by total numbers of oocytes obtained and numbers of embryos transferred. We therefore analysed oocyte utilisation rates using corrected MII numbers (see Methods) for three outcomes—production of usable embryos, clinical pregnancy and live-birth. Since oocyte quality is the principal determinant of embryo quality, we also analysed implantation rates and live-birth rates per embryo transferred as measures of per-embryo performance.

From a total of 319 and 256 MIIs produced by GV+ and GV− patients, respectively, fertilisation rates were 65.2% and 65.6% (*p* = 0.49) (Table 4). This resulted in a total of 74 and 75 usable embryos and slightly higher oocyte utilisation rates for GV− compared with GV+ oocytes (29.3% vs. 23.2%; *p* = 0.049) (Table 4). Notably, GV− patients also had significantly higher oocyte utilisation rates for clinical pregnancy (6.8% vs. 2.3%; *p* = 0.02) and live-birth (5.7% vs. 1.9%; *p* = 0.03). Furthermore, GV− patients also had 2–3 times higher rates of implantation (30.2% vs. 11.8%; *p* = 0.02) and live-birth per embryo transferred (25.6% vs. 9.8%; *p* = 0.04).

We were conscious that for IVF patients, oocytes were deemed to be immature on the day after TVOR at the time of the fertilisation check, whereas in the ICSI group, GV− oocytes were identified shortly after TVOR. Although only a minority of patients underwent IVF, it remained possible that the GV− group may have contained patients who had GV− oocytes on the day of TVOR that then subsequently underwent GVBD in the ensuing ~24 h and that this may have affected the outcomes. We therefore undertook a separate analysis restricted to ICSI cycles. Strikingly, the difference between the two groups become even more dramatic with markedly higher oocyte utilisation rates for clinical pregnancy (7.9% vs. 1.4%; *p* = 0.003) and live-birth (6.3% vs. 0.9%; *p* = 0.005) (Table 5). Furthermore, GV− patients also had around five times higher rates of implantation (33.3% vs. 7%; *p* = 0.005) and live-birth per embryo transferred (26.7% vs. 4.9%; *p* = 0.009) (Table 5).

### 3.3. DNA Damage Levels in Human GV− Oocytes Are Uniformly High

Next, we sought a molecular readout of oocyte quality. γH2AX accumulates at sites of DNA breaks, and the extent of γH2AX staining is directly proportional to the severity of DNA damage [23]. We therefore quantified γH2AX levels using confocal microscopy in GV− oocytes. We undertook γH2AX immunostaining of 26 human GV− oocytes obtained from 17 patients that were part of the foregoing clinical analyses.

**Figure 1:**
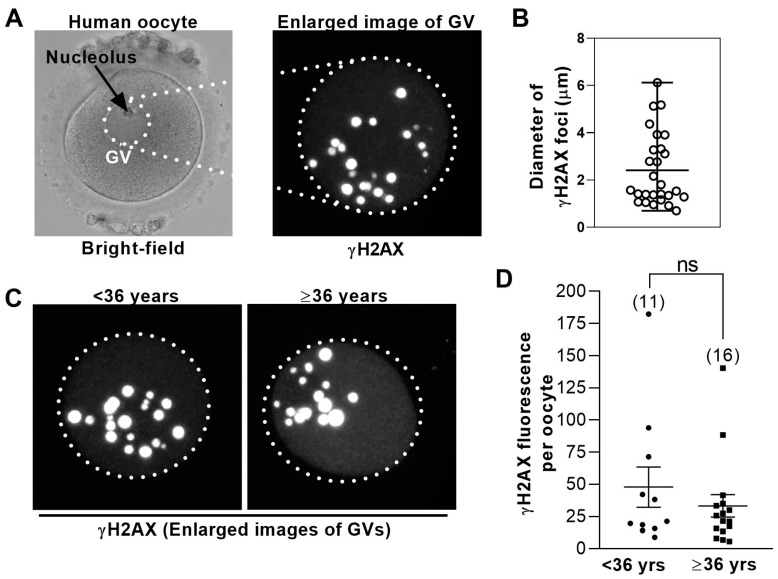
Levels of DNA damage in human germinal vesicle (GV) oocytes are not age-dependent. (**A**) Shown is a bright-field image of a GV− stage human oocyte (left); dotted circle highlights the GV. To the right is an enlarged view of the GV immunostained for γH2AX. Shown is a maximum projection of multiple z-slices. (**B**) The sizes of γH2AX foci in human GVs vary widely. The diameters of individual γH2AX foci from the oocyte in (A) were measured using LAS software and plotted. Bars represent the mean and range. (**C**) Shown is the representative γH2AX immunostained GVs from younger (<36 years of age) and older (≥36 years of age) women. Images are maximum projections of multiple z-slices. (**D**) Total γH2AX fluorescence intensity within the GV was measured using confocal microscopy (see Methods) in oocytes from younger and older women (numbers of oocytes are shown in parenthesis) and plotted for each oocyte. Bars represent mean and SD.

For investigating DNA damage in human oocytes, previous analyses either counted the number of γH2AX-positive foci [2] or scored oocytes as either positive or negative for γH2AX without quantification [14]. We observed that the size of γH2AX foci varied widely and that some foci were very large (Figure 1A,B and Supplementary Video S1). Since one large focus could potentially represent the confluence of multiple smaller foci at different confocal z-planes that become partially superimposed on one another when all z-planes are overlaid [24], we were concerned that simply counting foci would not fully evaluate the extent of damage. For this reason, we assessed total γH2AX fluorescence that took into consideration both foci number and size.

Oocyte quality is well-known to decline with female age and undergoes a particularly marked decline between 35–37 years. Consequently, professional bodies such as the UK’s National Institute for Health and Care Excellence (NICE) consider 36 years a critical transition age in women [25]. Given that the vast majority of embryonic aneuploidy is derived from meiotic errors in oocytes, which correlate inversely with oocyte quality [26], embryonic aneuploidy rates provide a readout of oocyte quality. Since one of the largest analyses of human embryonic aneuploidy shows that aneuploidy rates remain relatively stable between 31–36 years but increase steeply beyond 36 years of age [27], we chose to compare oocytes from women <36 years with those from women ≥36 years. Significantly, there was no difference in mean γH2AX fluorescence when oocytes from younger patients (<36 years; 11 oocytes) were compared with oocytes from older patients (≥36 years; 15 oocytes) (Figure 1C,D).

Altogether, these data show that DNA damage in human oocytes obtained from IVF patients is not age-dependent. In other words, these data suggest that the presence of GVs during IVF may reflect an independent vulnerability to poor oocyte quality, distinct from the effects conferred by female age.

## 4. Discussion

Here we find that the occurrence of GV− oocytes appears to be part of a more widespread phenomenon affecting overall oocyte quality rather than merely being a coincidental occurrence. The rates of usable embryos generated per oocyte (26%) and live-birth oocyte utilisation for GV− patients (5.7%) that we found were similar to rates of 31% and 5% found previously in much larger studies [15]. Furthermore, the proportions of MII and immature oocytes that we found, ~85% and 15%, respectively, are also entirely consistent with published literature [7,8,9,10]. Collectively, this supports that although our study group was small, it was a representative population.

For the best-prognosis patients, live-birth oocyte utilisation rates are significantly higher than average [15,16], indicating that measures of per-oocyte performance provide a readout of oocyte quality. We found that compared with GV− patients, the clinical pregnancy and live-birth oocyte utilisation rates of GV+ patients were ~threefold lower, which is indicative of significantly inferior oocyte developmental competence. Entirely in keeping with this, the potential of embryos derived from GV+ oocytes was also inferior to those from GV− oocytes; implantation rates and live-birth per embryo transferred rates were both two to threefold lower for GV+ patients. When these analyses were restricted to ICSI cycles only, the superiority of the GV− group became even more marked arguing that inclusion of IVF patients did not contribute to the better performance of the GV− group we observed, and indeed, may have had the opposite effect. Thus, based on the relatively small sample size in this study, the potential of MII oocytes produced in cycles in which GVs occur appears remarkably lower than if GVs are absent, arguing that GVs may not be a random occurrence but could reflect a wider underlying problem. Our findings are therefore not consistent with small-follicle aspiration as the sole explanation for GV− oocytes since one would then expect companion MII oocytes to perform similarly to MII oocytes from GV− cycles. Furthermore, aspiration of small follicles does not necessarily yield immature oocytes since MII oocytes can be obtained from ~40% of follicles as small as 3–10 mm in diameter [11].

Since clinical outcome measures strongly indicated suboptimal oocyte quality in the GV+ population, we hypothesised that DNA damage, which could both induce meiotic GV− arrest and contribute to poorer pregnancy success, could be an important underlying molecular cause, albeit we stress, not the only one. We therefore studied DNA damage levels in GV− oocytes using γH2AX immunostaining, an established methodology for quantifying DNA breaks used previously in human oocytes [2,14,23]. Unlike prior studies on human oocytes that either quantified damage based on numbers of γH2AX foci [2] or assigned damage as being either detectable or not [14], we quantified total damage that factored in both numbers and size of γH2AX foci. We applied high-resolution confocal imaging that recently allowed us to undertake the most in-depth analyses to-date of post-anaphase-onset events in oocytes [22]. Interestingly, the severity of DNA damage did not exhibit a statistically significant age-related difference in human oocytes, consistent with previous findings based on a different methodological approach [14]. The lack of a significant ageing effect suggests that some patients may be inherently prone to generating poor quality oocytes at any age. It is not known what might predispose some patients to DNA damage; one theory is that it may reflect inherently suboptimal DNA repair capability [4] that could be further exacerbated by artificially high ovarian stimulation regimes. Overall, combined with markedly poorer clinical outcomes in GV+ patients, these cell-based findings strongly suggest that the presence of GV− oocytes at the time of oocyte retrieval is a red flag for a cohort of low-prognosis oocytes.

We propose that high DNA damage could be a key cause of poor oocyte quality in these cycles and that the occurrence of GVs is one consequence of this defect; for some oocytes, damage levels are sufficiently high to induce a meiotic arrest at the GV− stage despite an enforced hCG trigger. Entirely consistent with this, it has recently been suggested that higher levels of DNA damage predispose human oocytes to remain GV− arrested after 30 h of in vitro culture [14].

Most studies involving GV− oocytes arising from stimulated cycles have focused on the potential of such oocytes to undergo maturation in vitro and/or produce embryos and pregnancies. We are not aware of another prospective study that has compared the remaining cohort of companion mature oocytes in GV+ cycles to mature oocytes from cycles lacking any GV− oocytes. One study compared pregnancy outcomes for cycles having ≤2 GV− oocytes with those having >2 GVs [8]. This paper found no difference in clinical pregnancy or delivery rates between the two groups. This is consistent with our findings, which indicate that it is the presence of any GV− oocytes, even one (12 of 29 of our patients produced only a single GV− oocyte), rather than how many GVs are present, that signals poorer outcome. It has been reported that immature oocytes are more likely to occur with greater degrees of ovarian hyper-response [12]. Interestingly, oocyte utilisation rates for live-birth decrease significantly when oocyte yield is high (>15) [15]. Taken together, these studies link high oocyte yields with an increased chance of GV− oocytes as well as with lower oocyte potential, supporting the possibility that excessive ovarian stimulation could compromise oocyte quality.

A major drawback of our study was its small size. However, the differences in outcomes between the groups were compelling especially when only ICSI cycles were considered, and it will be important to extend this work to determine whether findings hold up for larger numbers of patients. We also note that whilst not attaining statistical significance, there was nevertheless a trend towards more MII oocytes retrieved in the GV+ group than in the GV− group, which may have affected the results. Another limitation was that not all oocytes were utilised. Although we corrected for incomplete MII utilisation in our analyses, it remains possible that the actual performance of those oocytes might have produced different results. This may be difficult to resolve in future studies as most patients (12 of 14 (85.7%)) who had unutilised embryos had had a live-birth and may not therefore return for a sibling within the timeframe of a study, if at all.

Notable strengths of our study were complete outcome data for all pregnancies, its prospective nature and that both groups were well matched for key determinants of IVF outcome, including female age, mature MII oocyte yield as well as numbers and stage of embryos transferred. Highly significantly, in parallel with clinical analyses we also undertook molecular analyses of poor oocyte quality—DNA damage levels—not previously studied in the context of IVF outcomes, or to this depth, in human oocytes. Because our study suggests that the presence of GV− oocytes signals inferior per-oocyte and per-embryo potential, such an occurrence could be an important variable in deciding on numbers of embryos to transfer and may tip the balance in favour of multiple versus single embryo transfer.

## Figures and Tables

**Table 1:** Patient and treatment cycle characteristics.

	GV+	GV−	*p* Value
Number of patients	29	31	
Age (mean ± SD)	36.4 ± 4.8	35.4 ± 4.4	0.45
ICSI (%)	24 (82.8)	22 (71)	0.22
Total embryo transfer events	46	40	
Total embryos transferred (%)	51 (68.9)	43 (57.3)	0.1
Total embryos used (%)	55 (74.3)	52 (69.3)	0.25
Number of embryos per transfer (mean ± SD)	1.1 ± 0.3	1.1 ± 0.3	0.6
Single embryo transfers (%)	41 (89.1)	37 (92.5)	0.43
Blastocyst-stage transfers (%)	40 (78.4)	36 (83.7)	0.44
Complete embryo utilisation (%)	23 (79.3)	23 (74.2)	0.44

GV+: Patients with germinal vesicle-stage oocytes; GV−: Patients without germinal vesicle-stage oocytes.

**Table 2:** Infertility factors.

	GV+	GV−
Polycystic ovary syndrome (PCOS) (%)	3 (10)	4 (13)
Tubal factor (%)	6 (21)	5 (16)
Severe male factor (%)	6 (21)	5 (16)
Unexplained (%)	9 (31)	10 (32)
Endometriosis (%)	5 (17)	7 (23)

**Table 3:** Oocyte numbers and maturation stages.

	GV+	GV−	*p* Value
Total oocytes (GV + MI + MII)	405 (14 ± 7.4)	270 (8.7 ± 5)	0.002
Total GV− oocytes	70 (2.4 ± 2.0)		
Total MI-oocytes	16 (0.5 ± 0.7)	14 (0.4 ± 0.9)	0.65
Total MII-oocytes	319 (11.0 ± 6.9)	256 (8.3 ± 4.8)	0.08
Corrected MII-oocytes	259 (8.9 ± 6.1)	192 (6.18 ± 3.7)	0.04

Data in parenthesis are mean ± SD.

**Table 4:** Clinical outcomes.

	GV+	GV−	*p* Value
Fertilisation rates (%)	65.2	65.6	0.49
Number of usable embryos	74	75	
Oocyte utilisation rate–usable embryos (%)	23.2 (18.7–28.2)	29.3 (23.8–35.3)	0.049
Oocyte utilisation rate–clinical pregnancy (%)	2.3 (0.85–5)	6.8 (3.6–11.3)	0.02
Oocyte utilisation rate–live-birth (%)	1.9 (0.6–4.4)	5.7 (2.9–10.0)	0.03
Implantation rate (%)	11.8 (4.4–23.9)	30.2 (17.2–50.8)	0.02
Live-birth rate per embryo transferred (%)	9.8 (3.3–21.4)	25.6 (13.5–41.2)	0.04
Miscarriage rates (%)	16.7 (4.2–64.1)	15.4 (1.9–45.4)	0.47

Data in parenthesis are 95% CI.

**Table 5:** Clinical outcomes—ICSI cycles.

	GV+	GV−	*p* Value
Oocyte utilisation rate—clinical pregnancy (%)	1.4 (0.3–3.9)	7.9 (3.8–14)	0.003
Oocyte utilisation rate—live-birth (%)	0.9 (0.1–3.2)	6.3 (2.8–12.03)	0.005
Implantation rate (%)	7 (1.5–19.1)	33.3 (17.3–52.8)	0.005
Live-birth rate per embryo transferred (%)	4.9 (0.6–16.5)	26.7 (12.3–45.9)	0.009
Miscarriage rates (%)	33.3 (0.8–90.6)	20 (2.5–55.6)	0.32

Data in parenthesis are 95% CI.

## Supplementary Materials

The following are available online at https://www.mdpi.com/2076-3271/8/1/4/s1, Video S1: Confocal z-slices of human GV− oocyte immunostained for γH2AX. Shown is a movie of consecutive z-slices obtained with confocal imaging through the full depth of the GV of a human oocyte immunostained for γH2AX (red). All z-slices from this oocyte were projected to produce the image shown in Figure 1A (right).
